# Molecular mechanisms of metabolic dysfunction-associated steatotic liver disease (MASLD): functional analysis of glucose and fructose metabolism pathways

**DOI:** 10.1042/CS20257727

**Published:** 2025-11-06

**Authors:** Naila Rabbani, Paul J. Thornalley

**Affiliations:** 1 GloVitality (UK) Ltd, London1, U.K.; 2 College of Health and Life Sciences, Hamad Bin Khalifa University, Doha, P.O, 34110, Qatar

**Keywords:** metabolic dysfunction-associated steatotic liver disease (MASLD), insulin resistance, hepatic steatosis, glucokinase, carbohydrate response element binding protein (ChREBP), nuclear factor erythroid 2-related factor 2 (Nrf2)

## Abstract

Metabolic dysfunction–associated steatotic liver disease (MASLD) is a major non-communicable disease with global prevalence of 38% and no early-stage treatment. It has risk factors of insulin resistance, impaired glucose tolerance, type 2 diabetes, and diets rich in glucose and fructose. In this review, we explore evidence of abnormal increased early-stage glycolytic intermediates, glycolytic overload, in the initiation of MASLD and propose a new strategy for improved therapy. Glucose is mainly metabolized to glucose-6-phosphate by glucokinase (GCK) in the human liver. This is slowed by the competitive inhibitor, glucokinase regulatory protein (GKRP), with inhibition potentiated by fructose-6-phosphate and lifted by fructose-1-phosphate. The *in situ* activity of GCK is predicted to increase up to 3-fold by dietary glucose and over 4-fold with concurrent fructose. Related increased glycolytic intermediates activate carbohydrate response element binding protein (ChREBP), hexosamine pathway, and methylglyoxal-stimulated unfolded protein response (UPR). Activation of ChREBP contributes to enhanced lipogenesis and impaired suppression of hepatic glucose production by down-regulation of insulin receptor substrate-2 (IRS-2). IRS-1 signaling is maintained, contributing to enhanced lipogenesis through activation of sterol response element binding protein-1c and down-regulation of IRS-2. Hexosamine pathway activity stabilizes GCK and ChREBP to proteolysis, and the UPR stimulates inflammation and fibrosis. Hepatocytes then export glucose excessively, increasing fasting plasma glucose and risk of peripheral insulin resistance, type 2 diabetes, and vascular complications. Activators of nuclear factor erythroid 2-related factor 2 (Nrf2) provide a novel strategy for therapy. They divert excess glucose metabolism to the pentosephosphate pathway, decreasing activation of ChREBP and hexosamine pathway and formation of methylglyoxal, and decrease lipogenic gene expression. Nrf2 activator, *trans*-resveratrol and hesperetin combination, corrected glycolytic overload and insulin resistance clinically and now merits evaluation for early-stage treatment of MASLD.

## Introduction

Metabolic dysfunction–associated steatotic liver disease (MASLD) is a major non-communicable disease with global prevalence of 38% [1]. The prevalence of MASLD is higher, 65%, in patients with type 2 diabetes mellitus (T2DM) [2]. The heritability is 52%, indicating that both genetic and non-genetic risk factors are major influences on the development of MASLD [3]. MASLD has recently been redefined from the previous term of non-alcoholic fatty liver disease (NAFLD) with a greater emphasis on metabolic disturbance and diagnosis based on inclusion rather than exclusion criteria. Ninety-eight percent of subjects with NAFLD meet the diagnostic criteria for MASLD, and so these conditions have been considered as equivalent in assessing older literature for this review [4]. MASLD is diagnosed by evidence of hepatic steatosis, accumulation of lipid in >5% of hepatocytes, together with one or more cardiometabolic criteria: presence of overweight or obesity, impaired glucose tolerance or T2DM, hypertension, increased plasma triglycerides, or decreased high-density lipoprotein cholesterol [5]. Progression of MASLD to increased severity of symptoms leads to metabolic dysfunction-associated steatohepatitis (MASH), fibrosis, cirrhosis, and MASH-related hepatocellular carcinoma [5]. MASLD is also an independent risk factor for atherosclerotic cardiovascular disease (CVD)—reviewed in [6]. Indeed, CVD is the major cause of death of patients with MASLD [7]. Metabolic risk factors for the development of MASLD are: insulin resistance, impaired glucose tolerance and diabetes, and hypertriglyceridemia—particularly with imbalance between hepatic triglyceride production and clearance, and visceral adiposity [6]. In a large cohort study from the UK Biobank, the odds ratio for developing MASLD in subjects with T2DM compared with healthy control subjects was 2.6, corrected for other risk factors [8]. A meta-analysis of controlled trials of foods with added fructose found the odds ratio for developing hepatic steatosis was 1.7 [9]. This indicates that there are likely mechanistic factors linked to hepatic metabolism of glucose and fructose in the development of MASLD. The initiating mechanism of MASLD is considered below, particularly with respect to how the hypothesis of glycolytic overload may provide an improved explanation and guide to new safe and effective therapy [10].

## Glucose metabolism in hepatocytes

After ingestion of a meal, approximately one-third of the glucose absorbed into the portal vein is taken up and metabolized by the liver [11]. The major site of glucose metabolism in the liver occurs in hepatocytes, which represent ∼80% of the human liver by volume. Glucose is taken up into and exported from hepatocytes by the GLUT2 glucose transporter [12]. GLUT2 is a low-affinity glucose transporter (*K*_M_ for glucose = 17 mM) with high expression in hepatocytes. There is fast equilibration of glucose between the extracellular space and cytoplasm *in situ*. The rate of glucose metabolism in the hepatocytes is therefore controlled at the first step of glucose metabolism, rather than by membrane transport [13]. The first step of glucose metabolism in hepatocytes, formation of glucose-6-phosphate (G6P), is catalyzed by hexokinase-4 or glucokinase (GCK) with ATP cofactor [14]. Glucokinase has low affinity for glucose and sigmoidal kinetics, reflecting positive cooperativity; S_0_._5_ = 7.6 mM and Hill coefficient *n* = 1.7 [15]. The concentration of ATP in human hepatocytes is 2 mM [16] and *K*_M_ for ATP = 0.4 mM in the GCK-catalyzed step [15], such that GCK is always saturated and not limited by ATP cofactor. GCK activity increases in the absorptive phase and decreases in the fasting phase. The absorptive phase increase provides for increased glucose metabolism for glycogen synthesis. The fasting phase decrease prevents excessive recycling of glucose when it is being formed within hepatocytes for export. These rapid changes in activity of GCK are achieved through its interaction with an endogenous competitive inhibitor, glucokinase regulatory protein (GKRP) [17].

**Figure 1:**
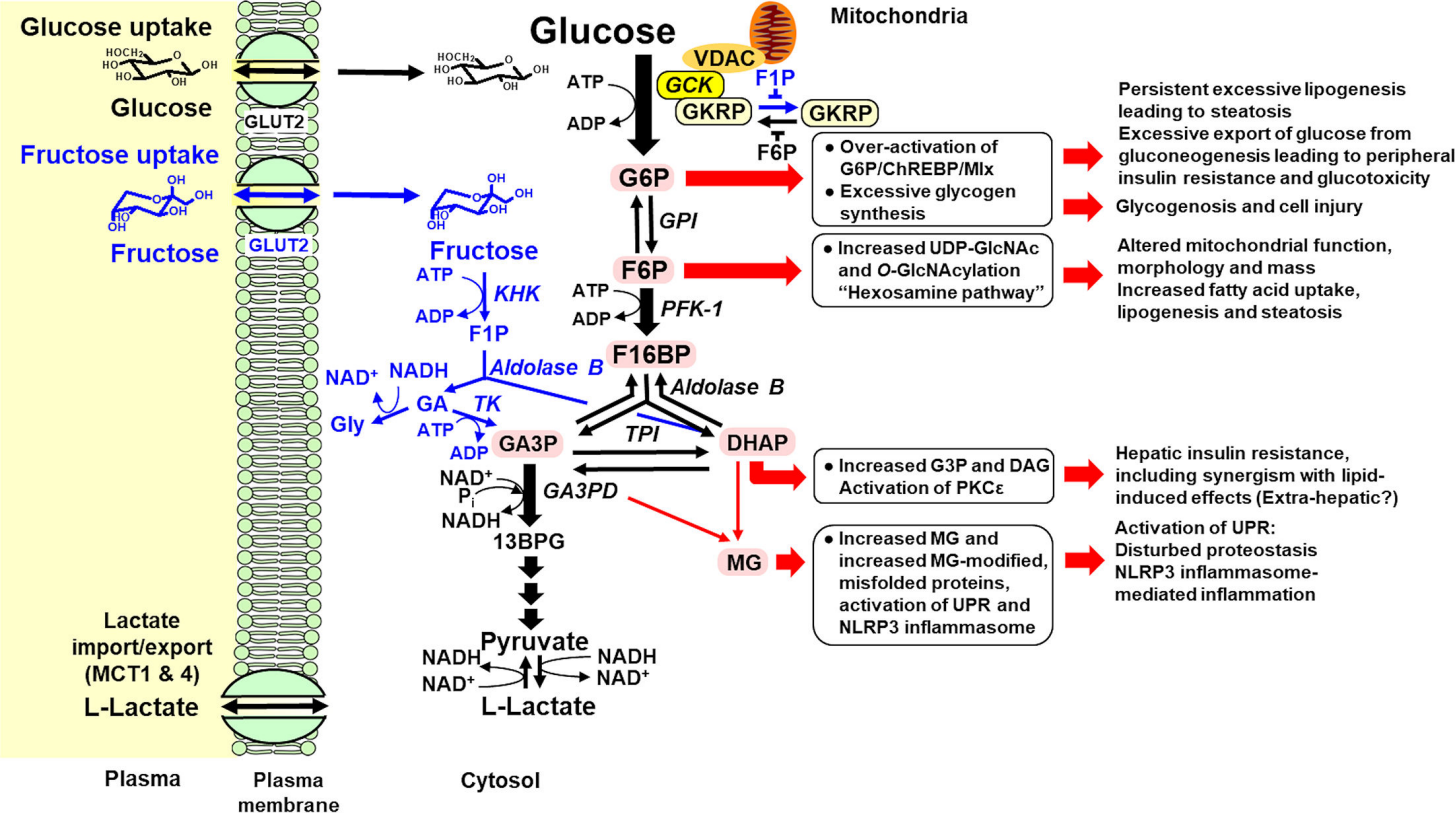
Unscheduled glycolysis and glycolytic overload in the initiation of metabolic dysfunction-associated steatotic liver disease. Key: pink back-filled metabolites, glycolytic intermediates increased in unscheduled glycolysis; red arrows: pathways of pathogenesis initiated by unscheduled glycolysis in hepatocytes; and blue metabolites and arrows: metabolism of fructose. Names of enzymes are given in italics. The nuclear translocation of GCK and GKRP is not shown for clarity.

GKRP binds human GCK with inhibitory constant K_i,GKRP_ = 984 nM [18]. The affinity is enhanced by the downstream metabolite, fructose-6-phosphate (F6P). In the presence of 200 µM F6P—the upper physiological limit, K_i,GKRP (+ 200 µM F6P)_ = 45 nM [19]. In the fasting phase with low plasma glucose concentration (3.5–5.5 mM), GCK is concentrated in the nucleus of hepatocytes bound to GKRP. The nucleus/cytosol concentration ratio of GCK and GKRP is ∼3.5 [20]. After a meal, the concentration of glucose in portal venous plasma increases to 7–11 mM and thereafter increases similarly in hepatocytes where corresponding glucose concentrations are ∼30% higher than in plasma [21]. Increased cellular glucose concentration displaces GCK from binding to GKRP in the nucleus, and GCK translocates to the cytosol, binding with mitochondrial voltage-dependent anion channel (VDAC) to receive ATP cofactor [22]. The nucleus/cytosol concentration ratio of GCK and GKRP decreases to ∼2.0 and 3.0, respectively [20]. In quantitative proteomics studies, the concentration of GCK and GKRP in human hepatocytes was 27 nM and 36 nM, respectively [23]. From enzyme kinetics calculations, it is estimated that from the fasting phase with FPG concentration of 4.5 mM to the absorptive phase of 7, 9, and 11 mM glucose, the *in situ* rate of the GCK-catalyzed metabolism of glucose increases by 1.9, 2.4, and 2.9-fold, respectively (Table 1). Therefore, as glucose concentration increases in the absorptive phase to levels typical of subjects with impaired glucose tolerance in prediabetes and fasting and absorptive phase of patients with T2DM, GCK-dependent metabolism of glucose continues to increase. Without an immediate increase in activity of downstream glycolytic enzymes, the steady-state concentrations of glycolytic intermediates in hepatocytes increase concomitantly. If this persists, it will likely be damaging [10] (Figure 1). Indeed, increased glucose exposure may be an independent risk factor for MASLD. Consistent with this, there was a nonlinear positive association of fasting plasma glucose (FPG) with risk of MASLD [24].

**Table 1:** Estimated relative in *situ* activity of glucokinase in human hepatocytes—effect of absorptive phase increase of glucose concentration, GKRP, F6P, and F1P

[Plasma glucose] (mM)	*r*_App,GCK (200 µM F6P)_ (fold change)	*r*_App,GCK (100 µM F1P)_ (fold change)
4.5	1.0	
7.0	1.9	3.4
9.0	2.4	4.0
11.0	2.9	4.4
4.5^GKRP-P446L^	1.6	
11.0^GKRP-P446L^	3.6	4.4
4.5^GKRP-R227Ter^	1.4	
11.0^GKRP-R227Ter^	3.6	4.4
7.0^GKRP↑2.0^	1.2	
11.0^GKRP↑2.0^	2.1	4.4

*r*_App,GCK (+200 µM F6P)_ and *r*_App,GCK (+100 µM F1P)_ are cellular mean apparent *in situ* activities of GCK in the presence of 200 µM F6P and 100 µM F1P, respectively—normalized to r_App,GCK (+200 µM F6P)_ with 4.5 mM glucose. Data for genetic variants of GKRP-P446L and GKRP-R227Ter are deduced assuming heterozygous mutation with only functional change for GKRP-P446L (wildtype levels of GCK and GKRP protein assumed) and GKRP-R227Ter represents a 50% loss of functional GKRP. Assumptions: cellular concentration of GCK and GKRP = 27 nM and 36 nM (or 18 nM in heterozygous GKRP null mutant, GKRP(-/+)) [23]; GKRP inhibitory constant in the presence of 200 µM F6P, K_i,GKRP (+200 µM F6P)_ = 45 nM; and in the presence of 100 µM F1P, K_i,GKRP (+100 µM F1P)_ = 7000 nM; for conversion of glucose to G6P by GCK, S_0_._5_ = 7.6 mM and Hill coefficient *n* = 1.7 [15]; nucleus/cytosol concentration ratio of GCK and GKRP is ∼3.5 with 4.5 mM glucose and 2.0 and 3.0, respectively, at higher glucose concentrations, with equal cytosol and nucleus volumes [20]; both nuclear and cytosolic GCK are catalytically active [20]; GKRP↑2.0 is 2-fold ChREBP induced expression of GKRP; and the hepatocyte/plasma glucose concentration ratio is 1.3. Michaelis-Mention equation used was: *r*_App,GCK =_ k_cat_[GCK][Glucose]*^n^*/(S_0.5_ [1+[GKRP]/K_i,GKRP_]*^n^* + [Glucose]*^n^*).

In summary, the flux of hepatic glucose metabolism is controlled by GCK and modulated by GKRP, shifting between low levels with GKRP inhibition in the fasting phase to higher levels with GKRP inhibition partially lifted in the absorptive phase. Sustained high glucose concentration drives excessive *in situ* activity of GCK, leading to the accumulation of glycolytic intermediates to abnormally high levels, which likely contributes to risk of MASLD.

## Fructose metabolism in hepatocytes

Fructose exposure originates mainly from the diet. There is also endogenous formation of fructose from glucose by the polyol pathway, but this accounts for only ∼10% of fructose exposure [25,26]. Fructose consumption in the USA was estimated as 55 g per day (range, 38–73 g per day) with about 30% of this from high fructose corn syrup (HFCS) [27]. Similar consumption was found in a European study with a lower proportion from HFCS [28]. Fructose is absorbed through the gastrointestinal tract epithelium on the luminal side by fructose transporter GLUT5 [29] and through the basolateral side by GLUT2 [30]. It is taken up by GLUT2 in hepatocytes and metabolized therein.

Recent studies in mice suggested that there may be significant metabolism of fructose in the intestines. At low doses of ingested fructose (<0.5 g/kg), 90% fructose metabolism occurred in the jejunum, duodenum, and ileum, with most of this converted to glucose and lactate and released into the portal circulation. At high doses of ingested fructose (≥1 g/kg), this pre-systemic metabolism was saturated and fructose was absorbed and metabolized in the liver and by colonic microbiota [31]. It is unclear how these doses relate to human subjects but the conversion of fructose to glucose by human jejunum has been found [32]. Nevertheless, consumption of 330 ml soda containing 40 g of HFCS (24 g fructose) increased plasma concentrations of fructose from 14 µM to a maximum of 150 µM in healthy control and obese subjects and to 220 µM in patients with T2DM [33]. This suggests fructose reaches the portal venous plasma at a clinical dose of 0.27 g/kg and thereby likely for most dietary fructose consumption. Dietary fructose, therefore, may be absorbed and taken up by the liver in the absorptive period [33].

 Fructose is metabolized in the liver to fructose-1-phosphate (F1P) by ketohexokinase (KHK) with ATP cofactor. KHK has a low *K*_M_ (800 µM) for fructose and high expression and activity in the hepatocytes [34,35]. F1P is an inhibitor of the binding of GCK with GKRP [18] and thereby increases the *in situ* activity of GCK. In the presence of saturating levels of F1P (100 µM), the affinity of GKRP decreases; K_i,GKRP (+100 µM F1P)_ = 7000 nM [18]. At this level of F1P, the *in situ* rate of glucose metabolism by GCK in hepatocytes is increased by 3.4–4.4 fold in the absorptive phase with 7–11 mM plasma glucose, compared with normal FPG (Table 1). Onward metabolism of F1P involves cleavage to dihydroxyacetonephosphate (DHAP) and glyceraldehyde by aldolase B (ALDOB), with glyceraldehyde converted to glyceraldehyde-3-phosphate (GA3P) by triokinase to supplement the triosephosphate pool of glycolysis and by alcohol dehydrogenase to glycerol for enhanced lipogenesis [36] (Figure 1).

After a meal, the liver takes up 33% of the oral glucose load and 70% of the oral fructose load. So, for typical daily consumption of 174 g sugar (∼54 g fructose and 120 g glucose) [37], similar amounts of glucose and fructose (∼40 g) are metabolized per day by the liver. This indicates there is efficient metabolism of fructose rate-controlled by KHK in hepatocytes. Studies from enzyme activity measurements in extracts of liver suggested the capacity of KHK to phosphorylate fructose is comparable with the capacity of GCK to phosphorylate glucose in rodent livers and 10-fold higher in human livers [38]. Stable isotopic tracer studies—providing more robust insight of *in situ* kinetics—suggested a 2.5-fold higher rate of hepatic metabolism of fructose than glucose in mice and 10-fold higher in human subjects [39,40]. In healthy human subjects, the hepatic concentration of F1P maximized rapidly at 3 min and returned to baseline values within 10 min after a 200 mg/kg bolus intravenous injection of fructose [41]. Diurnal variation of plasma fructose concentration in healthy control subjects showed increases from ∼20 µM in the fasting phase to 300–600 µM over 30–60 min periods at mealtimes, with individual variability of peak plasma concentration timing and magnitude. The half-life for return of plasma fructose to fasting levels after meals was ∼30 min [42]. In comparison, plasma glucose concentration increased from 5 mM to 7 mM at mealtimes with a similar half-life of return to fasting levels in healthy human subjects. This is influenced by insulin, glucagon, incretins, and other factors regulating changes in tissue uptake, metabolism, and formation of glucose [43]. From plasma to hepatocytes, there is a positive gradient of ∼1.3 for glucose concentration and a steep negative gradient of approx. −30 for fructose concentration [21,44]. This reflects that hepatocytes take up and metabolize glucose and also produce and secrete it, whereas they only take up and metabolize fructose. In the liver, fructose is mainly oxidized and converted to glucose, with little converted directly to lipids [40]. Nevertheless, hepatic fructose metabolism increased the deposition of glycogen [45], *de novo* lipogenesis, and induced hepatic insulin resistance [46]. A major contributing mechanism is likely lifting of the GKRP inhibition of GCK by F1P producing hepatocyte glycolytic overload—with increased G6P stimulating glycogen synthesis and G6P and fructose-2,6-bisphosphate (F26BP) activating carbohydrate response element binding protein (ChREBP) contributing to lipogenesis and insulin resistance—see below.

In summary, fructose is absorbed efficiently, extracted from circulation mainly by the liver, and metabolized rapidly by KHK. Its metabolite F1P activates GCK, driving excess hepatic glucose metabolism and glycolytic overload. While fructose itself is mostly oxidized or converted to glucose, its regulatory effects promote glycogen deposition, lipogenesis, and insulin resistance, linking high dietary fructose intake to increased risk of MASLD.

## Link of increased glucose and fructose metabolism to hepatic insulin resistance in MASLD—down-regulation of IRS-2 producing mixed insulin resistance

With normal hepatic insulin sensitivity, increased plasma insulin in the absorptive phase produces increasing insulin binding and activation of hepatocyte insulin receptor to regulate glucose and lipid metabolism. Increased insulin receptor occupancy triggers receptor autophosphorylation with recruitment and phosphorylation of insulin receptor substrates, IRS-1 and IRS-2. Phosphorylated IRS proteins then recruit and activate phosphoinositide 3-kinase (PI3K), which phosphorylates phosphatidylinositol 4,5-bisphosphate (PIP2) to form phosphatidylinositol-3,4,5-trisphosphate (PIP3). PIP3 recruits 3-phosphoinositide-dependent protein kinase 1 (PDK1) to activate Akt1 and Akt2 on Thr308/309 and Ser473/474, respectively, and downstream signaling involving mechanistic target of rapamycin complex-1 (mTORC1), glycogen synthase (GYS2), and the forkhead box protein O1 (FoxO1) transcription factors [47,48]. Akt2 is the major isoform, and Akt1 is the minor isoform in the liver. In mouse liver, Akt1 and Akt2 were 16% and 84% of total Akt, respectively, with no detectable Akt3 [49]. Akt2 is required for hepatic steatosis [50,51], whereas Akt1 is considered to have an important role in cell growth and likely basal lipid maintenance [52]. Akt1 partially compensated for Akt2 in the suppression of absorptive phase gluconeogenesis in hepatic Ak2 gene deletion experiments, suggesting also a role in decreased hepatic glucose production (HGP) [53]. Insulin signaling also triggers the association of the liver-specific class II PI3K, γ-isoform, with small GTPase and activated Akt2 in early endosomes. This provides a sustained activation of Akt2 and signaling to activate glycogen synthase [54]. Insulin thereby increases *de novo* lipogenesis (DNL), triglyceride formation and secretion, and glycogen synthesis, and suppresses fatty acid oxidation and HGP.

Signaling through IRS-1 increases lipogenic signaling through Akt2 and mTORC1-dependent and independent pathways to activate sterol response element binding protein-1c (SREBP-1c) [55]. Concurrent glucose metabolism-linked activation of ChREBP [56,57] and oxysterol-dependent activation of liver X receptor-α (LXRα) are required for maximum effect [58,59]. Hepatic expression of LXRα was also increased four-fold in patients with MASLD and correlated with increased expression of SREBP-1c [60].

 Signaling through IRS-2 mediates insulin-dependent increase of glycogen synthesis and decreased glycogenolysis and gluconeogenesis [61]. Akt2 signals through glycogen synthase kinase-3 (GSK3) dependent and independent pathways to activate GYS2 and suppress glycogenolysis [53,62], and through FoxO1 to decrease activities of glucose-6-phosphatase (G6PC) and phosphoenolpyruvate carboxykinase (PCK1) to suppress gluconeogenesis [63]. The insulin-dependent suppression of lipolysis in white adipose tissue (WAT) leads to decreased release and hepatic metabolism of non-esterified fatty acid (NEFA). This decreases hepatocyte pyruvate carboxylase activity through allosteric regulation by acetyl CoA [64]. HGP is thereby decreased in the absorptive period.

Hepatic insulin resistance is a major driver in the development of MASLD [65]. It is defined as the raised basal HGP in the presence of normal or raised plasma insulin levels [66]. Hepatic insulin resistance in MASLD has characteristics of *mixed* insulin resistance: preserved insulin-dependent lipogenic signaling and impaired insulin-dependent suppression of HGP [67]. In patients with MASLD and MASH, hepatic expression of IRS-1 was maintained, whereas expression of IRS-2 was decreased and correlated negatively with expression of PCK1 and G6PC [68]. Liver-specific knockout of IRS-2 expression in mice showed increased G6PC expression and impaired glycogen synthesis, including with high-fat diet (HFD) feeding [69]. How might the expression of IRS-2 be selectively decreased?

**Figure 2:**
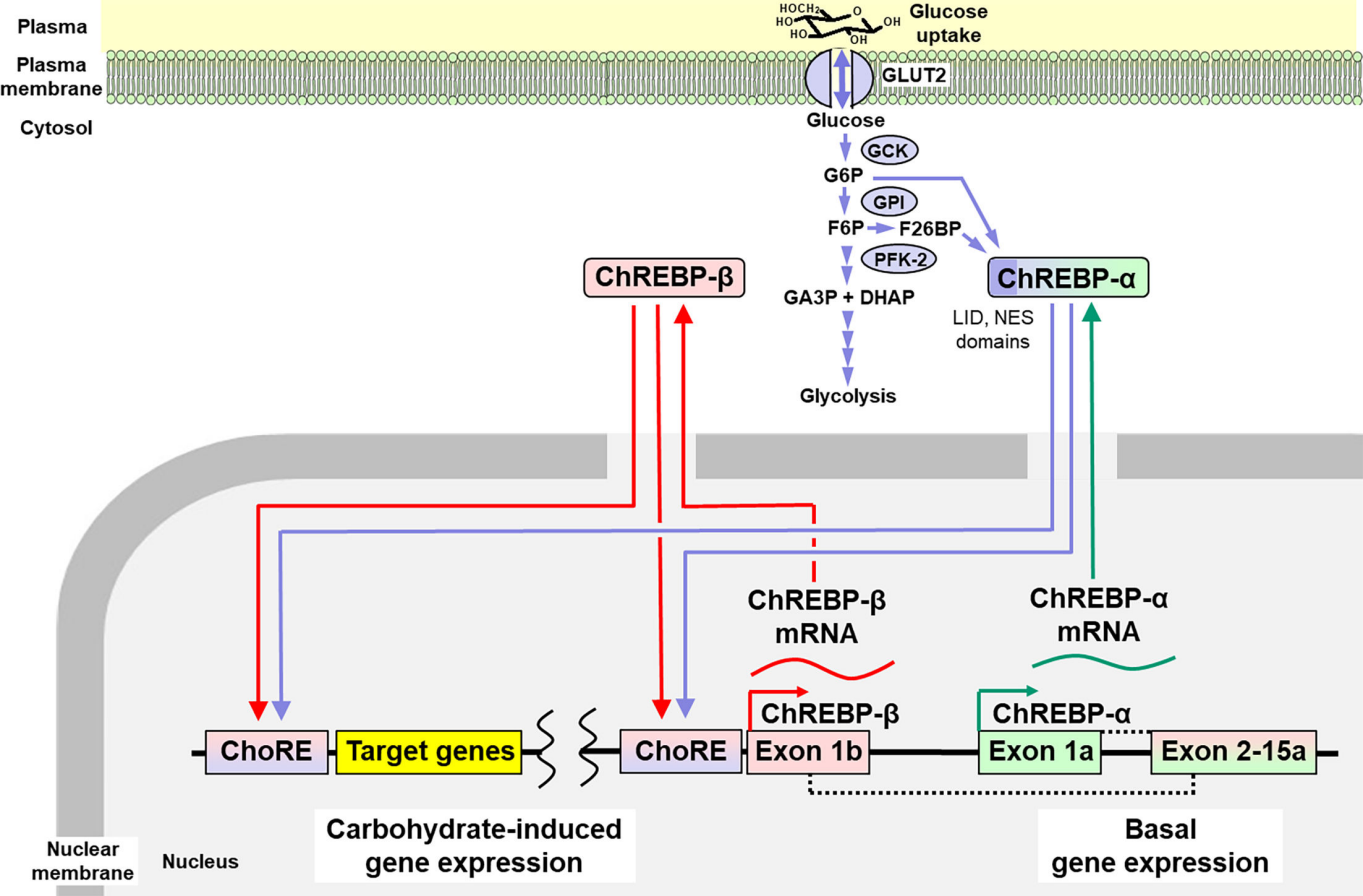
**Activation of ChREBP by glycolytic intermediates and regulation of carbohydrate response element-linked genes—including basal production of ChREBP-α and inducible production of ChREBP-β isoforms**. ChREBP-β is transcribed from an alternative first exon promoter 1b. The transcript is translated from exon 4, forming a shorter protein of 687 amino acids missing NES and LID domains. The ChREBP-β isoform is directly regulated by ChREBP-α through a ChoRE in exon promoter 1b. Adapted from [78,79]. Color key: green - basal expression of ChREBP-α; purple - activation by G6P and F26BP and transcription regulation of ChoRE-linked genes by ChREBP-α; red - induced expression and transcription regulation of ChoRE-linked genes by ChREBP-β.

Some of the earliest metabolic effects of increased glucose consumption are increased hepatocyte concentrations of G6P, F6P, and F26BP. F26BP is an allosteric regulator of glycolysis formed from F6P by phosphofructokinase-2/fructose-2,6-bisphosphatase (PFK-2/FBPase-2) [70,71]. G6P and F26BP bind and activate transcriptional activity of two paralogous transcription factors, ChREBP and Mondo A—also known as Mlx-interacting protein-like and MLX-interacting protein, respectively [72,73]. They form a complex with Max-like protein X (Mlx). ChREBP is the dominant transcription factor of this type in the liver which has basal expression of the α-isoform, ChREBP-α, from exons 1a to 15a. Within exon 1 a, there are five evolutionarily conserved ‘mondo conserved regions’ (MCR) in the N-terminus of ChREBP and Mondo A. MCRs are similar in sequence and structure to G6P-binding sites of other G6P binding proteins and through binding G6P or F26BP produce an allosteric activatory effect [74]. These comprise a ‘glucose-sensing module’ composed of a ‘low-glucose inhibitory domain’ (LID) and a ‘glucose response activation conserved element’. This has been called the ‘glucose sensor’ function of ChREBP. There are also two nuclear export signals (NES) and a nuclear localization signal domain regulating its translocation between cytosol and nucleus [74,75]. With G6P or F26BP bound, ChREBP-α/Mlx translocates to the nucleus and binds regulatory carbohydrate response elements (ChoREs) in target genes to increase expression of mainly glycolytic and lipogenic proteins [73,76,77]. One of these is the ChREBP gene itself, which thereby induces expression of a second isoform, ChREBP-β (Figure 2).

ChREBP-β is expressed from an alternative promoter and is induced by ChREBP-α through ChoREs near the start of exon-1b and lacks the LID domain and nuclear export signals. It is mainly located in the cell nucleus and has much greater transcriptional activity than ChREBP-α [80]. Increased cellular G6P and F26BP concentrations activate ChREBP-α isoform, which then initiates a potent feed-forward loop where increased expression of ChREBP-β is capable of binding to the ChoREs on its own promoter, producing more ChREBP-β [81] (Figure 2). ChREBP-β expression is increased in the livers of mice with high glucose or fructose feeding [82] and in the liver of human subjects living with obesity and patients with diabetes [83,84].

Fructose also activates ChREBP through increasing formation of G6P and F26BP by F1P lifting the GKRP inhibition of GCK. In rats, dietary glucose and fructose increased hepatic expression of ChREBPβ with fructose producing higher ChREBP transcriptional activity than glucose [82,85]. Leptin-deficient (ob/ob) mice are an experimental model of obesity that exhibit hyperphagia, gain of body weight, and peripheral and hepatic insulin resistance [86]. In ob/ob obese mice, liver IRS-2 mRNA levels were lower and G6PC but not PCK1 mRNA levels were higher than those in WT mice. Knockdown of hepatic ChREBP normalized hepatic IRS-2 mRNA levels and suppressed G6PC mRNA levels to lower than those of WT or ChREBP-deficient mice [87]. This response may be mediated through binding to the ChoRE in the promoter of the IRS-2 gene although down-regulation functionality in response to activated ChREBP is to be confirmed [88]. This implicates increased hepatic glucose metabolism in the down-regulation of IRS-2 of hepatic insulin resistance linked to the development of MASLD.

**Figure 3:**
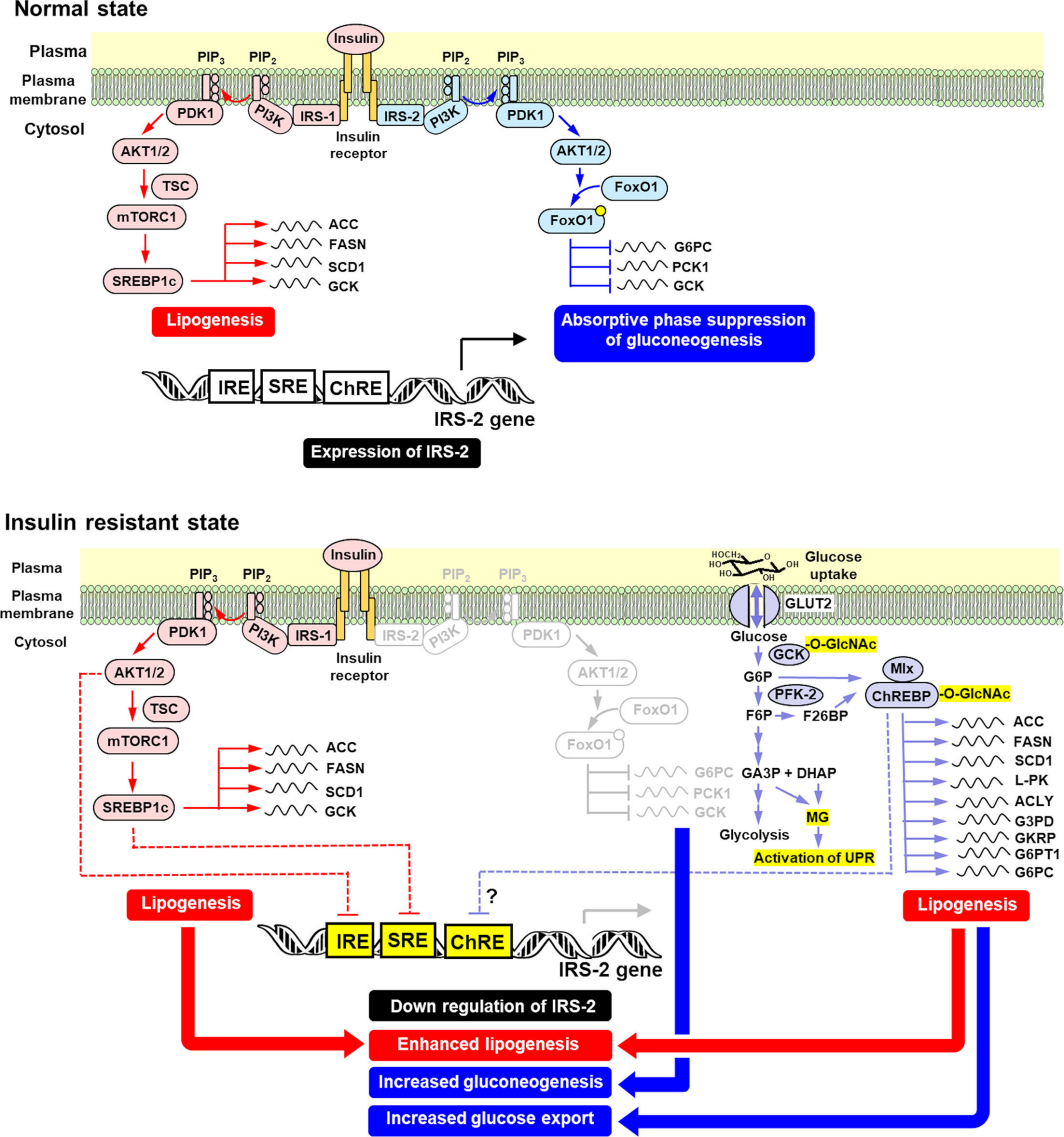
Mechanisms of hepatic insulin resistance and de novo lipogenesis in the development of MASLD discussed in the text. Greyed out pathway of IRS-2 indicates decreased down-regulation of gluconeogenesis and yellow highlighted IRS-2 promoter elements involved in down-regulation, hexosamine pathway O-GlcNAc modification of GCK and ChREBP and MG-linked activation of the UPR in MASLD—see text. Other contributory mechanisms are described in [92].

There are other contributing mechanisms to the down-regulation of IRS-2 expression. The IRS-2 gene has a functional insulin response element whereby insulin signaling via IRS-1 may repress the transcription of IRS-2 in a PI3K/Akt-dependent manner. This has been suggested as a contributory factor of decreased hepatic IRS-2 mRNA in chronic hyperinsulinemia [89,90]. This may also involve IRS-1 signaling through SREBP-1c and binding to sterol response element binding in the promoter of IRS-2 [91]. There was evidence of a multilayered epigenetic down-regulation of IRS-2 expression in patients with T2DM with hyperinsulinemia [91] (Figure 3). Extrahepatic mechanisms are also involved, including central nervous system and glucagon—reviewed in [62].

In summary, in healthy human subjects, insulin signaling - mainly via IRS-2/Akt2 - coordinates suppression of hepatic glucose output and promotion of glycogen and lipid storage. In MASLD, selective insulin resistance develops: IRS-1/lipogenic pathways remain active, while IRS-2/glucose-suppressive pathways are impaired. Excess glucose and fructose metabolism, via activation of ChREBP, contributes to IRS-2 down-regulation, hyperinsulinemia reinforces it, and together they drive development of hepatic insulin resistance and MASLD.

## Link of increased glucose and fructose metabolism to hepatic insulin resistance in MASLD—other supporting mechanisms

Increased flux of glucose metabolism in hepatocytes also increases the concentration of F6P. This activates the hexosamine pathway which increases protein O-linked β-N-acetylglucosamine (O-GlcNAc or O-GlcNAcylation) catalyzed by O-GlcNAc transferase. Hyperinsulinemia may also contribute to this response by increasing production of PIP3 which recruits O-GlcNAc transferase from the nucleus to the plasma membrane to catalyze O-GlcNAcylation [93]. GCK and ChREBP are targets of O-GlcNAcylation. Modification increases their stability to proteolysis, increasing hepatic glucose metabolism and ChREBP protein and transcriptional activity, respectively [94,95]. This synergizes with increased G6P and F26BP activation of ChREBP to potentiate the down-regulation of IRS-2 in MASLD. Fructose metabolism also activates the hexosamine pathway by increasing the formation of F6P through F1P lifting the inhibition of GCK by GKRP—see above.

Further downstream in early-stage glycolysis are the triosephosphates, DHAP and GA3P. Both triosephosphates have trace-level spontaneous degradation to the reactive glycating metabolite, methylglyoxal (MG) [96]. Increased triosephosphate concentrations produce increased formation and cellular concentration of MG, which increases the formation of the major advanced glycation end product, hydroimidazolone MG-H1 [97]. Formation of the hydrophobic MG-H1 moiety often occurs from positively charged hydrophilic arginine residues on the surface of proteins which thereby become misfolded [98]. MG-modified protein content of the liver was increased in HFD-fed rats [99] and in biopsies of patients with MASLD where increased MG was exacerbated by decreased glyoxalase 1 (Glo1) [100]—the major enzyme catalyzing the metabolism of MG [101]. MG-modified proteins are often misfolded and are the major stimulus for activation of the unfolded protein response (UPR) in high glucose concentration, activating all three ER-based sensors: inositol requiring enzyme-1α (IRE1α), protein kinase-like ER kinase (PERK), and activating transcription factor 6 (ATF6) [102]. IRE1α was activated in the liver of HFD-fed mice with insulin resistance and hyperinsulinemia, increasing splice variant transcription factor XBP1s. This interacts with the SREBP-1 promoter to potentiate the down-regulation of IRS-2 [103]. Activation of IRE1α also stimulates the activation of c-Jun amino-terminal kinase (JNK) [104] which contributed to decreased phosphorylation of IRS-1 and hepatic insulin resistance in high fat, high sucrose fed-mice [105]. Fructose may also activate the UPR through this mechanism by increasing the concentration of triosephosphates and thereby MG. This accounts for activation of IRE1α and JNK pathways in livers of high fructose diet-fed mice [106,107].

Increased flux through early-stage glycolysis in high glucose concentration increases the formation and cellular concentration of glycerol-3-phosphate (G3P) [70], leading to increased formation *de novo* of diacylglycerol (DAG) [10]. Hepatic insulin resistance in a background of increased lipid exposure was linked to increased concentration of hepatic DAG, activation of protein kinase c, ε-isoform (PKC_ε_). PKC_ε_ is highly expressed in liver and, when activated, produced an inhibitory phosphorylation of the insulin receptor at Thr1160 [108,109]. Increased DAG in the steatotic liver was proposed to originate from increased fatty acid availability through increased lipolysis in insulin-resistant adipose tissue of subjects living with overweight and obesity [109]. Treatment of HFD-fed rats with antisense oligonucleotide against PKC_ε_ protected against hepatic insulin resistance [110]. Increased fatty acid availability stimulating increased DAG and activation of PKC_ε_ was thereby proposed to contribute to hepatic insulin resistance and appeared to account for 64% of hepatic insulin sensitivity variability in human subjects living with obesity and without diabetes; markers of endoplasmic reticulum (ER) stress being also involved [111]. A further study, however, found liver-specific deletion of PKC_ε_ did not protect against HFD-induced insulin resistance, whereas global deletion of PKC_ε_ in liver, skeletal muscle, and adipose tissue did, implicating tissue cross-talk in HFD-induced hepatic insulin resistance. PKC_ε_-dependent changes in insulin receptor phosphorylation on Thr1177, corresponding to the site phosphorylated *in vitro* by PKC_ɛ_ [108], were not detected [112]. Mechanisms other than PKC_ε_ activation may be important in induction of hepatic insulin resistance in MASLD (Figure 3).

In summary, excessive hepatic glucose and fructose metabolism also drives insulin resistance through glycolytic overload-linked pathways: hexosamine pathway—stabilizing GCK and ChREBP to proteolysis, the latter suppressing expression of IRS-2; and increased MG with activation of the UPR. The contribution of DAG/PKCε signaling remains uncertain. Together, these processes contribute to mixed hepatic insulin resistance characteristic of MASLD.

## Link of increased glucose and fructose metabolism to steatosis in MASLD

Hepatic steatosis occurs when hepatocyte lipogenesis exceeds lipolysis and fatty acid oxidation. The origin of fatty acids of lipids deposited in hepatic steatosis of MASLD was: 59% NEFAs, 26% DNL, and 15% dietary origin [113]. Increased availability of NEFAs is associated with insulin resistance in WAT producing increased lipolysis [84]. Hepatic lipogenesis is stimulated by insulin-dependent signaling through IRS-1, Akt2, and mTORC-1 for expression of SREBP-1c and its lipogenic gene targets in hepatocytes. This is crucial for induction of lipogenic gene expression and steatosis [114]. Evidence in support of this is: (i) mice lacking the hepatocyte insulin receptor had marked insulin resistance and dysglycemia and did not develop MASLD even when challenged with a HFD [115]; and (ii) human subjects with mutations in the insulin receptor gene or inhibitory antibodies specific for the insulin receptor had insulin resistance and hyperglycemia without hepatic steatosis [114]. Insulin-dependent signaling through SREBP-1c also maintains expression of GCK and thereby susceptibility to increased hepatic glucose metabolism and glycolytic overload with sugar-rich diets [116].

Increased hepatic glucose metabolism stimulates lipogenesis through ChREBP activation by binding of G6P and F26BP [82]. Glucose-stimulated activation of ChREBP regulates expression of all enzyme genes involved in the DNL pathway of the liver, including liver-type pyruvate kinase (L-PK), acetyl-CoA carboxylase (ACC), fatty acid synthase (FASN), stearoyl-CoA desaturase-1 (SCD-1), ATP-citrate lyase (ACLY), and glycerol-3-phosphate dehydrogenase (G3PD) [117,118]. Indeed, deletion of hepatic ChREBP in mice decreased hepatic steatosis but increased hepatic insulin resistance and impaired glucose tolerance [119]. The latter two effects may be due to loss of ChREBP-induced increased expression of GKRP, G6PC, and G6P translocase (G6PT1). G6P translocase transports G6P from the cytosol to the lumen of the ER where G6PC resides. These gene expressions otherwise provide for decreased *in situ* metabolism of glucose in the absorptive phase and glucose export from hepatocytes [82,117,120]. Hepatocytes thereby are relieved of steatosis but experience excessive cellular glucose exposure and metabolism. Overexpression of hepatic ChREBP in mice increased lipogenic expression and hepatic steatosis [121]. In patients with MASLD, expression of ChREBP was increased with >50% hepatocyte steatosis [121]. This indicates that activation of ChREBP is a key mediator of hepatic steatosis and persistent, overactivation in glycolytic overload is the likely mechanistic cause.

Dietary fructose also stimulates lipogenesis in hepatocytes. Bolus consumption of 85 g fructose by healthy human subjects increased lipogenesis 2-fold greater than with equivalent consumption of glucose [122]. In a study of mice fed chow containing 30% starch + 3% sucrose or 60% starch or 60% fructose (fructose dose of 100–120 g/kg/day), both high starch and fructose diets increased hepatic steatosis by similar levels, but the fructose diet produced impaired glucose tolerance and more marked increased expression of ChREBPβ and glycolytic genes and lipogenic genes. The fructose diet induced increased expression of fructolytic genes (KHK, ALDOB, and triokinase) and gluconeogenic genes (G6PC and G6P translocase), stimulating the conversion of fructose to glucose. Activation of ChREBP by increased G6P and F26BP is implicated in these responses. These pathways may be operating clinically as liver biopsies from patients with MASLD showed positive correlation of ChREBPβ with FASN, L-PK, PCK1, and G6PC [82]. Fructose was considered to have greater lipogenic potential than glucose due to its metabolism via glyceraldehyde to glycerol [36]. However, the lifting of GKRP inhibition of GCK by F1P to increase glycolytic overload may also be involved—see above. The slower conversion of glyceraldehyde to GA3P by triokinase decreased overload of the triosephosphate pool, which was proposed as a cytoprotective response—albeit at the expense of increased steatosis [36] (Figure 3).

In summary, hepatic steatosis in MASLD is supported by increased NEFA influx and *de novo* lipogenesis, with insulin signaling through IRS-1 and SREBP-1c and glucose metabolism-linked activation of ChREBP activation as key regulators. Fructose is especially potent as it lifts GKRP inhibition of GCK to produce glycolytic overload with consequent strong activation of ChREBP. Persistent overactivation of these pathways drives the development and progression of hepatic steatosis.

## Role of hepatic unscheduled glycolysis and glycolytic overload in the initiation of MASLD

GCK-dependent glucose metabolism of hepatocytes confers susceptibility to unscheduled glycolysis and glycolytic overload [10]. Early-stage glycolytic intermediates consequently accumulate to abnormally high levels. Where the increases are persistent, multiple pathogenic processes are activated to damaging effect. Recent studies in mice provided experimental evidence of this occurring in the liver in response to high glucose intake. There was a sustained increase in metabolic flux through glycolysis—including also G3P which stimulates triglyceride and DAG synthesis, and high steady-state levels of early-stage glycolytic intermediates: G6P, F6P, and others. There was also a rapid and transient increase in F26P. The increases of G6P concentration and expression of ChREBP-linked genes, G6PC and PCK1, had high sensitivity with respect to dose of glucose [70]. Further supporting evidence of hepatic glycolytic overload came from studies of experimental obese, hyperglycemic leptin receptor mutant (fa/fa) rats with hepatic insulin resistance. At 12 weeks old, fa/fa rats had hepatic concentrations of G6P, F6P, GA3P, and DHAP increased two to three fold [123]. These abnormally high concentrations of early-stage glycolysis intermediates are linked to activation of pathogenic mechanisms of MASLD development (Table 2).

**Table 2:** Impact of increased glycolytic intermediates on hepatic insulin resistance and steatosis

MASLD feature	Glycolytic intermediate	Pathogenic effector mechanism	Reference
Hepatic insulin resistance	G6P (and F26BP)	Activation of ChREBP and increased expression of ChREBPβ, decreasing IRS-2	[73,77,82,85,87]
	F6P	Increased O-GlcNAcylation with increased ChREBP stability and transcriptional activity, decreasing IRS-2	[94]
	DHAP and GA3P	Increased MG, MG-modified proteins and activation of IRE1α and JNK, decreasing IRS-1 activity.	[99,100,105]
	DHAP	Increased G3P, *de novo* DAG formation, activation of PKC_ε_, and inhibitory phosphorylation of insulin receptor (extra-hepatic effect)	[70,108–112]
DNL and steatosis	G6P (and F26BP)	Activation of ChREBP, increased expression of ChREBPβ and lipogenic gene expression.	[82,117–119,121,124]
	F6P	Increased O-GlcNAcylation increasing (i) CD46 and lipid accumulation; and (ii) ChREBP stability and transcriptional activity.	[94,125]
	DHAP and GA3P	Increased MG, MG-modified proteins: (i) activation of IRE1α, increasing XBP1s interacting with the SREBP-1 promoter for increased lipogenic gene expression; and (ii) activation of PERK, increasing VLDLR and lipoprotein delivery to the liver.	[103,126]
	DHAP	Increased formation of G3P and triglyceride synthesis	[70]
Fibrosis	F6P	Increased O-GlcNAcylation correlates with increased fibrosis; mechanism unclear.	[127]
	DHAP and GA3P	Increased MG, MG-modified proteins with activation of IRE1α, increased TXNIP, and activation of the NLRP3 inflammasome	[128]

First, consider the effects of increased hepatocyte G6P. Incubation of hepatocytes with up to 10 mM glucose produced a relatively modest increase in cellular concentration of G6P (0.2 mM to 0.3 mM) [129]. This is mainly due to activation of liver GYS2 by G6P [130], which also inhibits glycogenolysis by inactivation of phosphorylase-*a* [129]. Glucose consumption increases as glycogen is produced [131]. Insulin also stimulates glycogen synthesis through activation of protein kinase B, which phosphorylates and inhibits GSK3, a negative regulator of GYS2 [62]. G6P interacts with an arginine-rich cluster in GSY2 where R582 is required for activation. With the R582A mutation, however, G6P-dependent activation of GSY2 is impaired and increased metabolic flux from G6P is directed into glycolysis and lipogenesis [132]. This indicates that when hepatic G6P is unusually high, glycolytic overload and lipogenesis ensue. Glycogen synthesis is also enhanced by increased F26BP, which is a cofactor for Mondo A/Mlx increased expression of protein targeting to glycogen (PTG)—a phosphorylase phosphatase increasing glycogen synthesis [133]. Abnormally increased glycogen deposition is found in MASLD associated with hepatocyte ballooning and cytotoxicity [134].

Increased G6P and F26BP activate transcriptional activity of ChREBP/Mlx in hepatocytes and, through this, increased expression of glycolytic, gluconeogenic, and lipogenic genes, which contributes to hepatic steatosis and insulin resistance—including increased expression and activity of ChREBPβ [73,77,82]. In support of this, lipogenic gene expression in patients with MASLD correlated positively with expression of GCK and ChREBPβ [82,124]. ChREBP activation provides for conversion of tricarboxylic acid cycle metabolites to fatty acids for energy storage, increased expression of G3PD for increased G3P formation for triglyceride synthesis [117]. A further important ChREBP-linked response is increased expression of GKRP, G6PC, and G6P translocase [117]. Increased expression of GKRP counters increased formation of G6P by inhibition of GCK; and G6PC and G6P translocase promote the conversion of G6P to glucose for its export from the liver.

Increased hepatocyte F6P is a precursor of F26BP and contributes to the activation of ChREBP. It also stimulates the hexosamine pathway and contributes to hepatic insulin resistance by increasing glycolytic overload and lipogenic responses by stabilizing GCK and ChREBP—see above. Increased O-GlcNAcylation in patients with MASLD was also linked to impaired mitochondrial function, increased fatty acid translocase/CD46 and lipid accumulation [125,127].

 Increased DHAP and GA3P produce increased concentrations of MG leading to increased formation of MG-modified proteins and activation of the UPR [102]. MG-modified protein content of the liver was increased in HFD-fed rats [99] and in biopsies of patients with MASLD where increased MG was exacerbated by decreased Glo1—the major enzyme catalyzing the metabolism of MG [101]. MG-modified, misfolded proteins activate the UPR [102]. Activation of IRE1α contributes to insulin resistance—see above, steatosis through interaction of XBP1s with the SREBP-1 promoter to increase lipogenic gene expression [103], and increases expression of thioredoxin interacting protein (TXNIP) which activates the NLRP3 inflammasome [102] contributing to inflammation and fibrosis [128,135]. Activation of the PERK pathway increased very low density lipoprotein-receptor (VLDLR) expression which increased lipoprotein delivery to the liver and steatosis [126]. ATF6 activation may have a protective role in MASLD by suppressing sterol regulatory element-binding protein-2 (SREBP2)-mediated lipogenesis and increasing fatty acid oxidation [136,137]. The rate of protein glycation by MG, however, has an *in situ* half-life of ∼6 h [138], so sustained exposure to increased MG concentration is required to produce these effects. Increased DHAP also leads to increased G3P which is linked to increased synthesis of triglycerides and DAG, contributing to hepatic insulin resistance and steatosis.

 High fructose feeding stimulates hepatic glucose-linked glycolytic overload by formation of F1P with countering of the inhibition of *in situ* activity GCK by GKRP and onward metabolism to increase the formation of triosephosphates and MG [82]. Clinical isocaloric restriction of dietary fructose decreased plasma D-lactate—a marker of flux of MG formation [139]. Fructose also induces expression of fructolytic enzymes through increased G6P and F26BP-linked activation of ChREBP, thereby increasing the rate of fructose metabolism in hepatocytes [82].

 In summary, excessive glucose metabolism and particularly fructose metabolism in hepatocytes drives glycolytic overload, leading to the accumulation of glycolytic intermediates to abnormally high levels driving cell dysfunction. These may lead to excessive glycogen deposition associated with hepatocyte ballooning and cytotoxicity, lipogenesis, increased protein O-GlcNAcylation, MG-driven protein misfolding, and ER stress. These contribute to hepatic steatosis, insulin resistance, inflammation, and fibrosis - core features of the development and progression of MASLD.

## Experimental and clinical evidence linking glucose and fructose-rich diets to steatosis, insulin resistance, and risk of MASLD

Many studies in the last two decades have investigated the effect of dietary intake of simple sugars—particularly glucose, fructose, and sucrose—on the decline of hepatic and metabolic health, including increased hepatic steatosis, DNL, and insulin resistance. Major dietary sources of sucrose are table sugar, cakes, and fruit [140]. In clinical studies, double-blinded, randomized controlled intervention trials provide strong evidence, particularly when outcomes of multiple studies are combined in meta-analysis—with due regard to the quality of the design and implementation of studies included in the analysis. Duration and dose of sugar intervention may also influence outcomes.

In a double-blinded, randomized controlled trial, subjects living with overweight and obesity consumed glucose- or fructose-sweetened beverages providing 25% energy requirements for 10 weeks. Hepatic DNL, FPG, insulin, and insulin resistance increased only in subjects consuming fructose [141]. Contrary to this, a short-term overfeeding study with fructose or glucose produced hepatic insulin resistance and increased hepatic fat content by both interventions [142]. Hepatic insulin resistance was induced in healthy subjects consuming sweetened beverages for 3 weeks with a dose of 80 g glucose or fructose per day; impairment of insulin-induced suppression of HGP was slightly greater with fructose compared with glucose [143]. A meta-analysis of 21 intervention studies found evidence that a diet supplemented by fructose or glucose increased liver steatosis [144]. In further meta-analysis of 65 intervention trials, there was evidence of increased hepatic steatosis with fructose and sugar-sweetened beverages [9,145]. A diet rich in simple sugars, therefore, increases steatosis.

There is also an increased risk from dietary saturated fat, which increased insulin resistance likely through increased ceramides [146]. In comparing the effects of overfeeding (1000 extra kcal/day) healthy overweight subjects for 3 weeks with saturated fat, unsaturated fat, or simple sugars, hepatic steatosis increased most (55%) with saturated fat and to less markedly with simple sugar (33%) and unsaturated fat (15%) [147]. The unsaturated fat in this study contained mainly MUFA and some polyunsaturated fatty acid (PUFA). Overfeeding lipids containing saturated fatty acids (SFAs) increased hepatic steatosis and also insulin resistance and endotoxemia [147]. SFAs activate the hepatic Toll-like receptor-4 (TLR-4), the main receptor for endotoxin, leading to activation of IκB kinase, up-regulating the *de novo* synthesis of ceramides, and ceramide-induced activation of protein phosphatase 2A. This inhibits insulin signaling by decreasing phosphorylation of Akt [148]. It also contributes to inflammation and progression to MASH [149]. A recent Mendelian randomization analysis and animal studies suggested hepatic steatosis is also associated with MUFA intake [150]. ChREBP increases the expression of SCD-1, which converts SFAs to MUFAs. This allows for safer hepatic storage with decreased risk of insulin resistance and steatohepatitis but still supports hepatic steatosis [121]. Replacement of SFA and MUFA with moderate intake of PUFA may decrease risk of hepatic steatosis [150].

There is a close link between hepatic steatosis and hepatic insulin resistance in the development of MASLD—reviewed in [151]. In HFD-fed rats, hepatic insulin resistance and steatosis developed within 3 days without insulin resistance in peripheral tissues (skeletal muscle and adipose tissue) and development of obesity [152]. Other studies have found that non-diabetic, normolipidemic subjects with MASLD have both hepatic insulin resistance and peripheral insulin resistance [65]. In studies in young, lean subjects with peripheral insulin resistance, increased physical exercise corrected muscle insulin resistance, which decreased net hepatic triglyceride synthesis, suggesting improved peripheral glucose disposal may slow the development of MASLD [153]. Genetic variants impairing insulin signaling, ectoenzyme nucleotide pyrophosphate phosphodiesterase-1, and IRS-1 are linked to increased risk of MASLD [154]. The interplay of hepatic and peripheral insulin resistance is expected as both contribute to the progression of MASLD. Hepatic insulin resistance contributes to increased FPG and development of peripheral insulin resistance [155,156].

In summary, experimental and clinical studies suggest excess simple sugars and saturated fats are key dietary drivers of MASLD, acting via DNL, ceramide pathways, and impaired insulin signaling. Fructose and SFAs are particularly harmful, while PUFAs are protective. Hepatic and peripheral insulin resistance may reinforce each other, increasing hepatic glucose and NEFA exposure to accelerate progression of MASLD.

## Why endogenous mechanisms fail to prevent hepatic glycolytic overload in simple sugar-rich diets and in hyperglycemia associated with prediabetes and diabetes

Normally, glycolytic overload is avoided by efficient disposal of G6P for glycogen synthesis, suppression of HGP, and increased expression of enzymes of early-stage glycolysis through insulin-dependent activation of PFK-2 to increase F26BP, and G6P and F26BP-linked activation of ChREBP-dependent glycolytic gene expression in the absorptive phase [73,117,157]. ChREBP-dependent increase of GKRP expression also slows the rate of glucose metabolism [117]. This is likely overwhelmed by diets rich in glucose and fructose and hyperglycemia. Expression of hepatic GCK is maintained through regulation by IRS-1 in hyperinsulinemia as insulin resistance develops [158] and decreased suppression by IRS-2-linked FoxO1 signaling [159]. The protective response of ChREBP-induced increased expression of GKRP [117] is predicted to be insufficient to normalize glucose metabolism when countered with high dietary fructose (Table 1).

 In high glucose concentration, GCK is increasingly detached from GKRP and susceptible to proteolysis [160]. In support of this, hepatic GCK expression was maintained at normal levels in patients with T2DM with good glycemic control (glycated hemoglobin A1C < 7.0%) and decreased 60% with poor glycemic control (A1C > 7.0%) [161]. However, the counteracting mass action effects of increased hepatic glucose concentration likely increased *in situ* activity of GCK in both of these patient groups.

In a further response to counter glycolytic overload, export of glucose is activated by ChREBP-linked increased expression of G6PC and G6P translocase [82,120]. In support of this, hepatic G6P levels increased two-fold with a regular diet and 3-fold with high starch and high fructose diets in ChREBP knockout mice [82,162]. This was proposed as a mechanism maintaining hepatic phosphate ester homeostasis [120] but also counters glycolytic overload in hepatocytes.

In summary, hepatocytes normally avoid potentially damaging accumulation of glycolytic intermediates through stimulation of glycogen synthesis and ChREBP-driven glucose export. High sugar diets and poor glycemic control overwhelm these defenses, keeping GCK activity high and driving glycolytic overload despite counter responses.

## Genetic variants of glucokinase regulatory protein and risk of MASLD

With the key regulatory role of GKRP in the control of glucose metabolism in hepatocytes, the clinical traits linked to genetic variants of GKRP gene may provide further insight into its important physiological role. A common genetic variant is rs1260326, with other variants, rs780093, rs780094, and rs3817588, in strong disequilibrium with it. rs1260326 is a non-synonymous variant substituting leucine for proline, P446L [163]. In a mouse model, P446L-GKRP had decreased stability and there were lower levels of GCK and GKRP proteins and GCK/GKRP complex in the nucleus. These features were replicated in human liver tissue from subjects homozygous for rs1260326 [164]. P446L-GKRP protein bound and inhibited GCK activity similar to the wildtype GKRP, with lifting of the inhibition by F1P. However, the potentiation of GKRP binding to GCK by F6P was decreased [17]. rs1260326 and rs780094 have similar traits: increased risk of MASLD and progression to MASH with increased plasma triglycerides, LDL, VLDL, and 2 h plasma glucose in an oral glucose tolerance test (OGTT) and decreased FPG, fasting insulin, and homeostatic model assessment of insulin resistance (HOMA-IR). There is also a rare variant with premature termination of transcription and expression of a non-functional protein (Table 3). For the P446L-GKRP protein, the predicted effect on the *in situ* kinetics of GCK in heterozygotes is a 1.6 fold increase in hepatocyte glucose metabolism in the fasting phase and moderate increase above wildtype at high glucose concentration (2.9 vs 3.6 fold increase). For the p.Arg227Ter, the effect is slightly lower in the fasting phase, a 1.4 fold increase, and a simlar moderate increase at high glucose concentration (2.9 vs 3.6 fold increase) (Table 1). The traits provide support for increased glucose metabolism in hepatocytes driving increased lipogenesis and steatosis with also increased secretion of triglycerides. The concomitant decrease in FPG is thereby expected to decrease the development of peripheral insulin resistance and risk of developing T2DM. The increased 2 h plasma glucose in OGTT was considered to be a consequence of decreased hepatic GCK linked to decreased proteolytic instability in the presence of decreased GKRP [167].

**Table 3:** Genetic polymorphism of glucokinase regulatory protein in MASLD.

Snp id (position)	Effected allele	Effected allele frequency	Effects on traits	Reference
rs1260326 (2:27508073)	T	Caucasians: 45% African Americans: 13% Hispanics: 36%	P446L polymorphism. increased steatosis, triglycerides, and VLDL; decreased fasting glucose, insulin, and HOMA-IR;	[165,166]
rs780094 (2:27594741)	T	Caucasians 40% African Americans: 18%	Located in intron. Increased liver steatosis and fibrosis, LDL, triglycerides, 2 hour plasma glucose, and risk of MASLD; decreased fasting glucose, insulin, and HOMA-IR. In strong linkage disequilibrium with rs1260326 and rs3817588	[167–170]
rs780093 (2:27519736)	T	Caucasians 46%	Increased triglycerides and decreased FPG. In strong linkage disequilibrium with rs780094 and rs1260326	[171,172]
rs3817588 (2: 27584716)	T	Caucasians: 30%	Located in intron. Decreased risk of type 2 diabetes. In strong linkage disequilibrium with rs780094.	[170]
rs149847328 (2:27503548)	T	Caucasians: ≈ 0.1%	R227Ter - stop codon at position 227 producing a non-functional protein. Case report developed NASH but was not found in a further heterozygote case identified	[173,174]

 It is surprising that decreased GKRP activity does not lead to increased unscheduled glycolysis, glycolytic overload, and development of hepatic insulin resistance producing increased FPG. We interpret this as with constant decreased expression and activity of GKRP, there is chronic exposure of hepatocytes to increased G6P, inducing increased transcription of glycolytic enzymes through G6P/Mondo A/Mlx and thereby countering unscheduled glycolysis while increased lipogenesis is sustained. Periodic unscheduled glycolysis induced by meals rich in simple sugars is more damaging because the metabolic challenge comes when the expression of glycolytic enzymes is relatively low and, therefore, the risk of glycolytic overload is consequently high. A similar effect was found for peripheral insulin resistance in mice with transgenic overexpression of hexokinase-2 (HK2) on a HFD but not in mice with concurrent overexpression of GLUT1 and HK2 [10]. This prediction is deserving of experimental verification.

 In summary, GKRP variants such as P446L chronically enhance hepatocyte glucose metabolism, increasing lipogenesis and risk of MASLD while paradoxically lowering fasting glucose and insulin resistance risk. Hepatic gene expression may adapt by persistent up-regulation of glycolytic enzymes to prevent glycolytic overload. In the wildtype, the liver is particularly vulnerable to glycolytic overload because acute high sugar loads are received when the expression of glycolytic enzymes is relatively low.

## Insight into therapeutic intervention from the glycolytic overload hypothesis

From the viewpoint of glycolytic overload-stimulated hepatic insulin resistance and steatosis, a strategy for pharmacotherapy is the development of GCK inhibitors. This was discussed by Ashcroft *et al*. [175] with respect to preventing decline of pancreatic β-cell function in glucotoxicity—also linked to GCK-linked glycolytic overload [10]. Insight into the physiological effect of GCK inhibition may be gained from homozygous and heterozygous deletion of the GCK gene. Homozygous global deletion of GCK produced mice which died four days after birth from severe diabetes and severe liver steatosis. The latter is likely a consequence of the lack of insulin secretion and increased mobilization of NEFA from lipolysis in WAT. Heterozygous global GCK deletion produced mice with basal plasma glucose concentration increased by 60% at two days and 74% at six to ten weeks, decreased hepatic glycogen, mild hepatic steatosis, and impaired glucose tolerance and rate of hepatic glycogen synthesis, compared with wildtype controls. Liver-specific deletion of GCK had a much milder phenotype: with <5% hepatic GCK activity of controls, basal plasma glucose concentration was increased only 10% whereas basal plasma insulin was increased two-fold; liver and muscle glycogen content were normal but the rate of hepatic glycogen synthesis was decreased 90% [176]. Heterozygous loss-of-function mutations of GCK in human subjects produce monogenic diabetes, a subtype of maturity onset diabetes of the young (MODY) with plasma glucose increased 30–40% and impaired hepatic glycogen synthesis [177–179]. The effect of decreased GCK expression in the liver is partly countered by increased glucose concentration such that the *in situ* GCK activity in hepatocytes is expected to be similar or slightly lower than that of wildtype controls. Treatment of the mild hyperglycemia is not recommended outside pregnancy because glucose-lowering therapy is considered ineffective and the risk of vascular complications is considered similar to that in the general population [180,181].

An alternative approach is to target increasing the affinity of GKRP for GCK for enhanced *in situ* inhibition of GCK in hepatocytes. To our knowledge, small molecule GKRP enhancers have not been developed and may be ineffective for fructose-rich diets.

A strategy proposed to counter the adverse effects of high fructose diets is the development of KHK inhibitors. Studies in mice with HFD feeding with 30% fructose in drinking water for ten weeks found knockdown of KHK expression by siRNA and KHK inhibitor, PF-06835919, led to partial improvement of liver steatosis. Knockdown of KHK decreased *de novo* lipogenesis, decreasing hepatic fructose metabolism and improving glucose tolerance. PF-06835919 only partially decreased hepatic fructose metabolism and also targeted triokinase leading to the accumulation of F1P, glycogen accumulation, hepatomegaly, and impaired glucose tolerance [44]. In the clinical trial, it showed weak effectiveness in improvement of hepatic steatosis of patients with T2DM and MASLD [182] and was dropped from further clinical development for undisclosed reasons. Other candidate KHK inhibitor development remains in progress [183].

A likely fruitful approach is small molecule activators of transcription factor, nuclear factor erythroid 2-related factor 2 (Nrf2), which regulates the expression of ∼1300 cytoprotective genes with one or more functional antioxidant response elements (AREs) [184]. A multifactorial beneficial effect is expected: increased expression of glucose-6-phosphate dehydrogenase (G6PD) to divert accumulating G6P to the pentosephosphate pathway (PPP) [185,186]; increased expression of Glo1 to prevent activation of the UPR [102,187]; and decreased expression of SREBP1, ACC, and FASN, producing an anti-lipogenic response [188].

The hepatic PPP normally accounts for ∼16% glucose metabolism [189,190]. The NADP^+^/NADPH redox state influences *in situ* activity of G6PD when the reaction is in rapid dynamic equilibrium. Then high demand for NADPH pulls the equilibrium over the products, NADPH + 6-phosphogluconate, as demand for NADPH increases—as in the neutrophil respiratory burst, for example [191]. Metabolic flux studies of the liver indicated the G6PD-catalyzed reaction is not at equilibrium but rather under kinetic control of the forward reaction [192], metabolomics studies indicated there was no significant change in NADP^+^/NADH ratio in the steatotic liver [193] and the G6PD-catalyzed reaction is saturated by G6P substrate; K_M,G6P_ = 54 µM [194] and the cellular concentration of G6P ≈ 150 µM [192]. Therefore, increased cellular G6P alone cannot increase the metabolic flux through the PPP. Increased expression of G6PD is required, which may be achieved by activation of Nrf2 [186]. Concurrent activation of transketolase avoids increased cellular concentration of xylulose-5-phosphate, which has been proposed as an activator of ChREBP [195,196].

Nrf2 activation has been studied by both pharmacological and genetic activation. The latter is achieved by hypomorphic knockdown of the gene for Kelch-like ECH-associated protein 1 (Keap1)—an endogenous negative regulator of Nrf2 [188]. HFD-fed mice with homozygous deletion of Nrf2 developed MASLD, MASH, and cirrhosis, with greater induction of lipogenic genes than HFD-fed wildtype controls, suggesting endogenous Nrf2 responses are protective [197]. In HFD-fed mice, genetic activation of Nrf2 slowed increase in body weight, improved glucose tolerance, and decreased hepatic steatosis. This was associated with decreased fasting plasma glucose and insulin, decreased HGP and hepatic expression of G6PC, PCK1, and FASN [198].

Pharmacological activators of Nrf2 have been identified in functional assays. This is required rather than specific receptor targeting as the mechanism of Nrf2 activation remains uncertain. A mechanism involving increased translocational oscillation frequency of Nrf2 between cytosol and nucleus was proposed [185]. Investigation of induction of each target gene expression of interest is also required as Nrf2 activators have differing ARE-linked gene expression responses. Synthetic Nrf2 activators had activity against MASLD in experimental models [199,200]. Off-target effects may, however, produce serious adverse clinical effects, such as in the development of Bardoxolone methyl for treatment of diabetic kidney disease [201].

We reasoned that for long-term treatment and prevention of MASLD, where often health is already compromised by diabetes, highly tolerated and effective Nrf2 activators are required. A source of these is dietary bioactive compounds. We developed and optimized Nrf2 nutraceutical activators for Nrf2-dependent induction of Glo1 expression with application for prevention and treatment of insulin resistance. Screening for both Nrf2-dependent induction of expression of ARE-linked genes, quinone reductase-1 (NQO1) and Glo1, we found optimum Glo1 gene expression was achieved with a combination of *trans*-resveratrol and hesperetin (tRES + HESP; also called GlucoRegulate). In a randomized, double-blind, placebo-controlled cross-over clinical trial in overweight and obese subjects, treatment with tRES + HESP (capsules containing 90 mg *trans*-resveratrol and 120 mg hesperetin, once daily) for 8 weeks decreased FPG and postprandial glucose. It also corrected insulin resistance as assessed by the oral glucose insulin sensitivity (OGIS) index [202]. Interestingly, OGIS is a diagnostic marker of both hepatic fibrosis and steatohepatitis [203]. The increase in OGIS with tRES + HESP treatment was + 42 ml/min/m^-2^ for highly overweight and obese subjects (BMI ≥ 27.5 kg/m^2^) and + 58 mlmin^-1^m^-2^ in obese subjects (BMI ≥ 30.0 kg/m^2^); compare + 62 mlmin^-1^m^-2^ following 23 kg weight loss after gastric band surgery in severe obesity [204] and carbohydrate restricted diet and high-intensity interval training, + 19 mlmin^-1^m^-2^ [205]. This supplement may be suitable for treatment of early-stage MASLD—defined as hepatic steatosis with up to moderate fibrosis, stage 3 (F3). This is considered potentially reversible and where non-pharmacological therapy and pharmacological therapy are advised [206]. Current recommendations for treatment of early-stage MASLD are 3–5% loss of body weight, an improved diet (for example, a Mediterranean diet) and increased physical exercise ( > 150 min/week moderate intensity physical activity) [207] and with Resmetirom, a selective thyroid hormone receptor β agonist, for subjects with MASH of fibrosis stages 2 and 3 (F2 and F3) [208] and glucagon-like peptide-1 receptor antagonist, semaglutide [209]. Both pharmacotherapies have potential adverse effects where a highly tolerated dietary supplement would offer advantages [206,210]. In gene expression analysis of peripheral blood mononuclear cells, tRES + HESP treatment decreased expression of monocyte chemoattractant protein-1, interleukin-8, and receptor for advanced glycation end products [202,211]. This anti-inflammatory effect may prevent fibrosis and progression of MASLD to MASH [212–214]. tRES + HESP was well tolerated with high compliance to treatment [202]. There is enhanced clinical pharmacological effect by combining tRES with HESP on glycemic control and insulin resistance, likely through synergism in activation of Nrf2 and HESP improving the bioavailability of tRES [202,215]. tRES alone was ineffective in clinical evaluation for treatment of MASLD [216].

The expected benefit of RES + HESP treatment of MASLD is correction of hepatic insulin resistance, steatosis, and inflammation. This may be achieved by down-regulation of lipogenic gene expression and prevention of hepatic glycolytic overload, ChREBP, and UPR responses. There is also expected benefit from improvement of peripheral insulin resistance [202], and potentially countering the impaired incretin effect for improved glucose disposal and appetite control [210]. tRES + HESP is a candidate dietary supplement treatment for clinical MASLD.

In summary, pharmacotherapy for early-stage MASLD may target GCK, GKRP, KHK, or Nrf2. Of these, Nrf2 activation by nutraceutical GlucoRegulate currently shows the best balance of efficacy, safety, and tolerability, making it a promising adjunct to lifestyle interventions for early MASLD.

## Concluding remarks

The liver has a unique role in glucose metabolism in the body, providing for glucose storage in the absorptive phase and glucose production and export in the fasting phase while exposed to intermittent high glucose and fructose dietary inputs. Abnormal magnitude and persistence of increased early-stage glycolytic intermediates in hepatocytes is likely damaging to glucose and lipid homeostasis—explaining why hepatocytes uniquely have *in situ* inhibition of GCK by GKRP. Dietary fructose is particularly damaging because it lifts GKRP inhibition of GCK to enhance glycolytic overload and also induces fructolytic enzyme expression. A cytoprotective response is increasing hepatic export of glucose which extends into the fasting phase to increase FPG. While protective for the liver, this shifts the risk of glycolytic overload also to skeletal muscle, adipose tissue, pancreatic beta-cells, vascular cells, and cells of the incretin effect. Long-term this contributes to the development of peripheral insulin resistance, impaired incretin effect, T2DM, and related vascular complications [10,210]—also partly explaining why MASLD is a risk factor for T2DM and CVD [6,217]. From the glycolytic overload hypothesis, a new and likely effective and safe strategy to counter MASLD has emerged: activation of Nrf2 by tRES + HESP (GlucoRegulate). Correction of insulin resistance in overweight and obese subjects in clinical trials suggests it may also be effective for treatment of early-stage MASLD, which is now deserving of evaluation.

## Open Access

This article has been published open access under our Subscribe to Open programme, made possible through the support of our subscribing institutions, learn more here: https://portlandpress.com/pages/open_access_options_and_prices#conditional

## Abbreviations

A1C: glycated hemoglobin HbA1c
ACC: acetyl-CoA carboxylase
ACLY: ATP-citrate lyase
AKT1/2: serine/threonine kinase 1/2 (protein kinase B beta)
ALDOB: aldolase B
ARE: antioxidant response element
ATF6: activating transcription factor 6
13BPG: 1,3-biphosphoglycerate
CVD: cardiovascular disease
ChREBP: carbohydrate response element binding protein
ChoRE: carbohydrate response element
DAG: diacylglycerol
DHAP: dihydroxyacetonephosphate
DNL: de novo lipogenesis
ER: endoplasmic reticulum
FASN: fatty acid synthase
F26BP: fructose-2,6-bisphosphate
F1P: fructose-1-phosphate
F6P: fructose-6-phosphate
FPG: fasting plasma glucose
FoxO1: forkhead box protein O1
GA: glyceraldehyde
GA3P: glyceraldehyde-3-phosphate
GA3PD: glyceraldehyde-3-phosphate dehydrogenase
GCK: glucokinase
GKRP: glucose kinase regulatory protein
GLUT2: glucose transporter type 2
GLUT5: glucose transporter type 5 (fructose transporter)
G3P: glycerol-3-phosphate
G6P: glucose-6-phosphate
G6PC: glucose-6-phosphatase
G3PD: glycerol-3-phosphate dehydrogenase
G6PD: glucose-6-phosphate dehydrogenase
GPI: glucose-6-phosphate isomerase
G6PT1: glucose-6-phosphate translocase
GSK3: glycogen synthase kinase-3
GYS2: glycogen synthase
Glo1: glyoxalase 1
Gly: glycerol
HESP: hesperetin
HFCS: high fructose corn syrup
HFD: high fat diet
HGP: hepatic glucose production
IRE: insulin response element
IRE1α: inositol requiring enzyme-1α
IRS-1: insulin receptor substrate-1
IRS-2: insulin receptor substrate-2
JNK: c-Jun amino-terminal kinase
KHK: ketohexokinase
Keap1: Kelch-like ECH-associated protein 1
LID: low-glucose inhibitory domain
L-PK: liver-type pyruvate kinase
LXRα: liver X receptor, α-isoform
MASH: metabolic dysfunction-associated steatohepatitis
MASLD: metabolic dysfunction–associated steatotic liver disease
MCR: mondo conserved regions
MCT: monocarboxylate transporters
MG: methylglyoxal
MG-H1: methylglyoxal-derived hydroimidazolone, isomer-1
MODY: maturity onset diabetes of the young
MUFA: monounsaturated fatty acid
Mlx: Max-like protein X
NAFLD: non-alcoholic fatty liver disease
NEFA: non-esterified fatty acid
NES: nuclear export signal
NLRP3: NOD-, LRR-, and pyrin domain-containing protein 3
NQO1: quinone reductase-1
Nrf2: nuclear factor erythroid 2-related factor 2
OGIS: oral glucose insulin sensitivity
O-GlcNAc: O-linked β-N-acetylglucosamine
O-GlcNAcylation: O-linked N-acetylglucosamine moiety modification of ser and thr residues of proteins
PCK1: phosphoenolpyruvate carboxykinase
PDK1: 3-phosphoinositide-dependent protein kinase 1
PERK: protein kinase-like ER kinase
PFK-1: phosphofructokinase-1
PFK-2/FBPase-2: phosphofructokinase-2/fructose-2,6-bisphosphatase
PIP2: phosphatidylinositol 4,5-bisphosphate
PIP3: phosphatidylinositol-3,4,5-trisphosphate
PKCε: protein kinase c, ε-isoform
PPP: pentosephosphate pathway
PTG: protein targeting to glycogen
PUFA: polyunsaturated fatty acid
SCD-1: stearoyl-CoA desaturase-1
SFA: saturated fatty acid
SRE: sterol response element
SREBP-1c: sterol response element binding protein-1c
T2DM: type 2 diabetes mellitus
TK: triokinase
TLR-4: Toll-like receptor-4
TPI: triosephosphate isomerase
TSC: tuberous sclerosis complex
TXNIP: thioredoxin interacting protein
UDP-GlcNAc: uridine diphosphate N-acetylglucosamine
UPR: unfolded protein response
VDAC: voltage-dependent anion channel
VLDLR: very low density lipoprotein-receptor
XBP1s: spliced form of X-box binding protein 1
mTORC1: mechanistic target of rapamycin complex-1
tRES: trans-resveratrol
